# Relationship between Career Motivation and Perceived Spiritual Leadership in Health Professional Educators: A Correlational Study in Iran

**DOI:** 10.5539/gjhs.v6n2p145

**Published:** 2013-12-20

**Authors:** Jamil Sadeghifar, Mohammadkarim Bahadori, Donia Baldacchino, Mehdi Raadabadi, Mehdi Jafari

**Affiliations:** 1 Dept. of Health Services Management, School of Health Management and Information Sciences, Iran University of Medical Sciences, Tehran, Iran; 2 Health Management Research Center, Baqiyatallah University of Medical Sciences, Tehran, Iran; 3 Faculty of Health Sciences, University of Malta, Msida, Malta; 4 Research Center for Health Services Management, Institute for Futures Studies in Health, Kerman University of Medical Sciences, Kerman, Iran; 5 Students Scientific Research Center (SSRC), Tehran University of Medical Sciences, Tehran, Iran; 6 Health Management and Economics Sciences Research Center, Iran University of Medical Sciences, Tehran, Iran; 7 School of Health Management and Information Sciences, Iran University of Medical Sciences, Tehran, Iran

**Keywords:** spiritual leadership, career motivation, health professionals, educators, Iran

## Abstract

**Background and Aims:**

Career motivation in university educators through efficient ways and appropriate with the educational system, is considered one of the important factors affecting education of students and their competence. This study aimed to determine the relationship between career motivation and spiritual leadership among a university of medical sciences in the west, Iran.

**Methods:**

This descriptive, cross-sectional correlation study was conducted among the university educators of medical sciences in the west, Iran in 2012. All of the educators (N=230) were selected and recruited according to census method. The data were collected by two established self-completed questionnaires on spiritual leadership (SL) and career motivation.

**Data were analyzed statistically by parametric tests:**

Pearson correlation, independent student t-test and one-way analysis of variance (ANOVA).

**Results:**

The Pearson correlation test identified a significant relationship between educators’ career motivation and vision, altruistic love, hope/faith, meaning/calling and membership dimensions of spiritual leadership (p<0.05). The independent t-test detected a significant relationship between the ‘hope/faith’ (p=0.04) and organizational commitment (p=0.004) dimensions and the gender of educators. ANOVA revealed significant differences in educators’ years of work experience and their overall career motivation (p=0.003) and the dimension of ‘membership’ (p<0.04). A significant relationship was found in ‘altruistic love’ and ‘Hope/faith’, and the educators’ academic rank place in the university (p=0.03). Also a significant relationship was found in ‘vision’ (p=0.03) and ‘altruistic love’ (p=0.002) and ‘membership’ (p=0.04) dimensions, and the type of faculty.

**Conclusion:**

The results indicate that the dimensions of existence of spiritual leadership may have a positive relationship with educators’ career motivation.

## 1. Introduction

In the present era, with economic growth and technological changes, some organizations adopted the necessary changes to create a favorable environment to attract, maintain and motivate employees to improve their performance (Franceschini & Turina, 2013; Geijsel, Sleegers, Leithwood, & Jantzi, 2003). Factors such as, job dissatisfaction, lack of trust in organizations, global economic crisis, and increase in unemployment, demonstrated that rational actions of organizations to satisfy the demands of employees was unsuccessful. This yielded the need for enrichment in the work environment and meaningful work (Cullen, Edwards, Casper, & Gue, 2013; Lips-Wiersma & Wright, 2012). Thus, the absence of the spiritual dimension and the lack of attention to the holistic perspective of the employees as a holistic person generated the importance of the concept of spirituality in the workplace (Pawar, 2009; Gupta, Kumar, & Singh, 2013).

Spirituality is an attitude or a lifestyle that recognizes the dimensions associated with spirituality. Religion is a specific style of using spirituality, which is very often associated with an institutional religious affiliation. Spirituality was defined as being highly personal; refers to all religious beliefs and related patterns of behavior and emotions; and may be oriented towards a higher power and or/God (Rahimi, 2013; Dent, Higgins, & Wharff, 2005). Recent research studies results showed that religion is mainly related to the official-organizational religion. Additionally, spirituality is often associated with a feeling of closeness to God and the universe and living things. Religion is more focused on specific groups and organizations, while spirituality is broader which may even involve more than one religion (Reave, 2005).

Spirituality is a proven framework of organizational values in the respective culture that develops the experience of staff in transcendence through the work process, and facilitates their sense of connection with others in a manner that will provide them with a sense of completeness and peace (Daniel, 2010). Spirituality at work is an inspiring and motivating force for constantly searching for meaning and purpose in the work life, personal understanding of value of work and personal belief system. Spirituality should be seen as an integral characteristic of leaders in today’s organizations. This inseparability is a factor which contributes towards self- development in order to achieve higher levels of internal and external work success. Thus, public awareness on the need of inclusion of spirituality in education and at work appears to be on the increase (Aydin & Ceylan, 2009).

Spirituality and leadership are both complex concepts. A positive relationship is found between spirituality at work and spiritual in the theories and recent research on spirituality (Al Arkoubi, 2008; Fry, 2013).

The realization of this concept is crucial, especially in the higher education which is one of the most important aspects of sustainable development. This is because the education system needs healthy, happy and highly motivated educators. However, after a few years of working, educators reported to face numerous problems in the workplace and fatigue due to job stress and sometimes they desire to withdraw from their work (Davodi & Oshtori, 2011; Gregory, 2012). Hence, the higher education centers need to have managers who link their personal values and educators values to organizational values which may eventually enhance motivation to develop educational contexts and stimulate growth. Therefore, the role of spiritual leadership is to create such a spiritual environment. This is because they have an important role in the group and work effectiveness which may generate enhancement of the organizational structures and satisfaction of the spiritual needs of the of staff members (Crossman, 2010; Neal, 2013).

### 1.1 Conceptual Theoretical Framework

The spiritual leadership theory has been developed by Fry (2003) within an intrinsic motivation model and is based on characteristics such as hope, faith and altruism, and its aim is creating homogeneous vision and values at the individual, team, and organizational level, which may eventually generate higher levels of commitment and productivity of organization (Fernando, Beale, & Geroy, 2009). Fry introduces seven dimensions of spiritual leadership:

#### 1.1.1 Vision

In the 1980s, the Vision is known as a matter of leadership literature, because the leaders are bound to pay more attention to the organization’s future because of intense global competition, shorter cycle of technology development and the strategies that are rapidly becoming obsolete by the competition. The vision reflects the Units destination and gives meaning to their aspirations, and encourages hope and faith. Vision refers to the picture of the future with a clear explanation of why people are trying to create such a future (Fry, Hannah, Noel, & Walumbwa, 2011).

#### 1.1.2 Altruistic Love

Altruistic Love or Altruism is set of values, assumptions and ways of thinking that are morally right, and are shared by group members and taught to new members (Fry et al., 2011).

#### 1.1.3 Hope/Faith

Belief is certainty and confidences in the things that we desire and relying things which are not seen. Faith firmly is to believe something for which there is no proof or evidence and physical and material existence don’t prove it (Fry et al., 2011). Hope and faith are the origin of this belief, that vision, goals and mission successfully will be achieved.

#### 1.1.4 Membership

Membership includes cultural and social structure that we are immersed in it. It is a sense of understanding and appreciation, which are stemming from the interaction and communication through social interaction and group membership (Fry et al., 2011).

#### 1.1.5 Meaning/Calling

Refers to a transcendent experience or how to create significant difference by serving others and thereby meaning and purpose in life is created. People not only seek the competence and knowledge of their job, but also seek for the feel that this work has a social meaning or value (Pfeiffer, 2003).

#### 1.1.6 Organizational Commitment

On organizational commitment, people are linked by the senses of meaning and membership and remain faithful to organizations and want to stay in organizations that their culture is based on values and altruism and (Fry, 2003).

#### 1.1.7 Productivity

The productivity is interpreted as intelligent work. This definition shows that it is sufficient that organizations works intelligent and get optimal benefit of resources, it is the best way to improve productivity. The continuous improvement means that we don’t reach the end in the productivity and the new ways to increase productivity will always be found by innovation (Fry et al., 2011).

**Figure 1:**
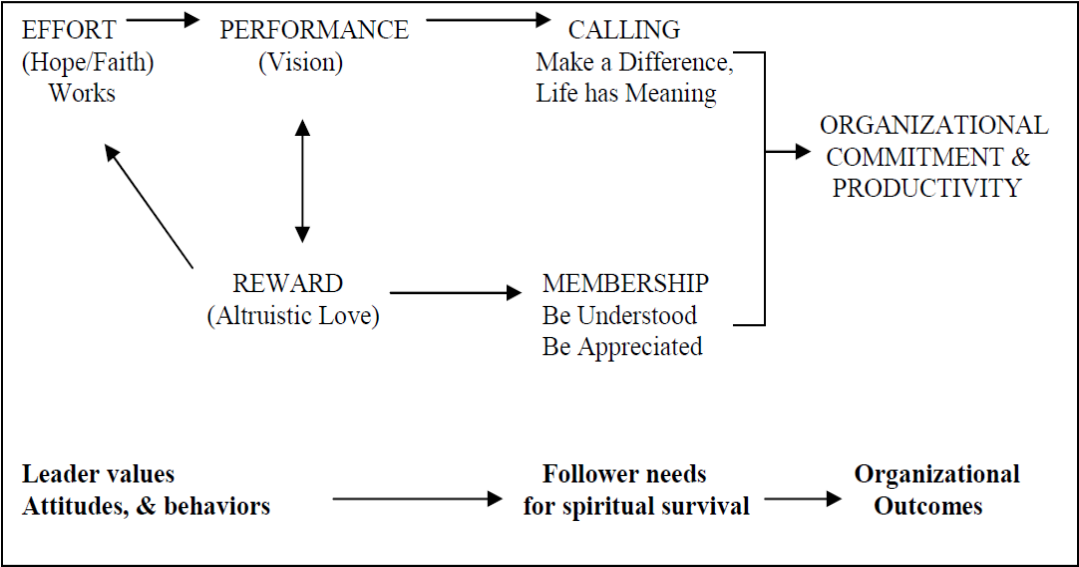
Causal model of spiritual leadership (Fry, 2003)

The leadership style of the managers who show interest in spirituality was found more effective than that of those managers who do not include spirituality in their work. Hence this infers a positive relationship between spirituality and leadership (Strack, Fottler, & Kilpatrick, 2008). Fry, Vitucci and Cedillo (2005) acknowledge this spiritual leadership model as a new and effective theory in the area of research on operational leadership, and suggests that the leader should emphasise more on the spiritual needs of the persons in the workplace in order to produce good organizational and individual results. Spiritual Leadership was found to have a positive significant influence on the spiritual well-being and organizational-personal performance variables by mapping faith to a shared vision and progress culture, which may eventually enhance the workers’ physical welfare and health (Fry & Nisiewicz, 2013). Therefore, more expectancies of spiritual leaders in various fields may increase the spirit of cooperation, trust, commitment and effectiveness of the organizational system (Mohammadi, Vanaki, & Mohammadi, 2012).

However, to achieve organizational objectives, the motivational factors of the staff members should be identified and facilitated. However, this may be difficult because of the individual differences in motivation and therefore, the characteristics of individuals should be assessed before any motivational action (Abdullah, 2010). Career motivation system was found as the most important factor in the success or failure at work and if neglected, wastage of enterprise resources may result. Torabi found that only 20% of the factors of success depend on the staff members` effort, whereas 80% may depend on their motivation. Currently, job motivation creates a challenge for organizations who recruit staff members without that motivation (Torabi, 2007). Thus, motivation appears to be the most important factor that sets the organization in competitive advantage and may help staff members to achieve organizational goals and stay focused on the job with better performance (Al-Rfou & Trawneh, 2009). Also, factors such as, lack of job security, working long hours, low salaries and inadequate resources may reduce employees’ job motivation (Khuwaja, Qureshi, Andrades, Fatmi, & Khuwaja, 2003). Therefore, assessment of the current level of motivation and spiritual leadership of the employees and managers respectively, may enhance progress and may help to overcome barriers.

The current research gap is diminishing of motivation among educators in medical sciences universities and poor efforts rarely by cash incentives for enhancing their motivation. It seems for sustainable motivation should be concentrated on inner and non-cash incentives. Thus, this study aimed to investigate the relationship between career motivation and spiritual leadership among educators of a university of medical sciences in the west of Iran in 2012.

## 2. Methods

### 2.1 Sample

The target population was the educators working in a university of medical sciences in the west of Iran in 2012. A list of educators in mentioned university was available and to guarantee greater representation of the data, all of the educators (N=230) as a sample were selected by census method. From the target sample of 230 questionnaires, 217 questionnaires were completed, 13 were discarded as incomplete. Hence, the final response rate was 94%. The final sample consists of 48 females (22%) and 169 males (78%); 84 working in medicine faculty (38.7%); 53 in public health (24.4%); 38 in allied medicine (17.5%); and 42 in Nursing & Midwifery (19.3%).

### 2.2 Measures

#### 2.2.1 Spiritual Leadership Questionnaire

The questionnaire on spiritual leadership (SL) of Fry et al (Fry, et al., 2005) was adapted to higher education for the purpose of this study. This questionnaire consisted of 33 statements in 7 dimensions including:

Vision (n=5) - describes the organization’s journey and why we are taking it; defines who we are and what we do. Sample items for vision dimension include “I understand and am committed to my organization’s vision”; “My organization’s vision inspires my best performance” and “I have faith in my organization’s vision for its employees”.
Hope/faith (n=5) - the assurance of things hoped for, the conviction that the organization’s vision/purpose/mission will be fulfilled. Sample items for Hope/faith are “I always do my best in my work because I have faith in my organization and its leaders” and “I set challenging goals for my work because I have faith in my organization and want us to succeed”.
Altruistic love (n=7) - a sense of wholeness, harmony, and well-being produced through care, concern, and appreciation for both self and others. Sample items for Altruistic love dimension are “My organization really cares about its people”; “My organization does not punish honest mistakes” and “The leaders in my organization are honest and without false pride”.
Meaning/calling (n=4) - a sense that one’s life has meaning and makes a difference. Sample items for meaning/calling dimension are “The work I do is very important to me”; “My job activities are personally meaningful to me”; “The work I do is meaningful to me”; and “The work I do makes a difference in people’s lives”.
Membership (n=5) - a sense that one is understood and appreciated. Sample items for membership dimension are “I feel my organization understands my concerns”; “I feel my organization appreciates me, and my work”; and “I feel my organization demonstrates respect for me, and my work”.
Organizational commitment (n=4) - the degree of loyalty or attachment to the organization. Sample items for organizational commitment dimension are “I would be very happy to spend the rest of my career with this organization”; and “I really feel as if my organization’s problems are my own”.
Productivity (n=3) - efficiency in producing results, benefits, or profits. Sample items for productivity dimension are “In my department, work quality is a high priority for all workers”; and “In my department, everyone gives his/her best efforts”.

#### 2.2.2 Career Motivation Questionnaire

The likert-form questionnaire was adapted from Niue Bachbr (1990) (Noe, Noe, & Bachhuber, 1990). This questionnaire consisted of 26 statements in 3 dimensions including:

Career insight (n=8) - Sample items for this dimension include “do you have a specific career goal?”; “do you have a specific plan for achieving your career goal?”; “do you feel you are aware of your skill strengths and weaknesses?”; and “do you ask co-workers you respect for Feedback on your performance?”.
Career identity (n=5) - Sample items for this dimension are “do you spend your free time on activities that will help your job?” and “have you taken courses toward a job-related degree?”
Career resilience (n=13) – some of Sample items for this dimension are “Do you accept compliments rather than discount them?”; “do you reward yourself when you complete a project?” and “have you designed better ways of doing your work?”

Both questionnaires underwent content validity by experts and were adapted according to the university structure and culture. Following modification of the instruments based on the experts’ recommendations, agreement was reached by the experts and researchers and the final version of both questionnaires was concluded. Reliability of the questionnaires was tested by the Cronbach alpha coefficient (Perceived Spiritual Leadership: 0.78; Career Motivation: 0.83). Both coefficients demonstrated the internal consistency and homogeneity of both questionnaires.

All the above scales utilized a 1–5 (from strongly disagree to strongly agree) response set. Individual scores were calculated by computing scale averages for each dimension.

### 2.3 Data Collection and Analysis

The participants were visited by the researchers at their work place who handed to them the questionnaires. In cases which the researchers did not have access to, the electronic version of questionnaire was sent to them by email. The researcher visited educators and collected questionnaires by hand, after one week. After one week, in some cases reminders were sent for educators.

Data were entered and processed using the Statistical Package for the Social Sciences (SPSS) software, the version 18. Following non-significant results of the Kolmogorov - Shmirnov test which demonstrated a normal distribution of the data of spiritual leadership and career motivation data, parametric tests were used such as, independent student t-test; one-way analysis of variance (ANOVA) and Pearson correlation coefficient. T-test/ANOVA identifies ‘differences in results’ whilst Pearson identifies relationships (without cause and effect).

### 2.4 Ethical Issues

According to the type of study, the researchers do not gain any formal ethical approval from the studied University’s Research Ethics Committee. The main ethical issues were respondents’ right to self-determination, anonymity and confidentiality. The questionnaires with a participant information sheet on the nature of the study were distributed to educators working in clinical and educational departments in the studied University. Normally, questionnaires need not a written consent form. So, the participants consented verbally. The return of the completed questionnaire demonstrates their consent to participate in the study. The questionnaire data were kept confidential and respondents were assured of their right to withdraw at any time. The names of the respondents were not recorded on the questionnaire, thus rendering the data anonymous

## 3. Results

From the total of 230 distributed questionnaires, 217 were returned rendering a response rate of 94%. Out of all respondents, almost all were male (169, 77.8%), and had assistant academic degree (107, 49.3%). The majority of respondents had 6-10 years of experiences (82, 37.7%), while less than 8.0% held more than 21 years of experiences (17, 7.8%). More respondents worked in medical faculty (84, 38.7%) compared with public health (53, 24.4%), allied medicine (38, 17.5%), and nursing and midwifery faculty (42, 19.3%) (Table 1).

**Table 1:** Demographic characteristics of participants

Variable		No	%
Gender	Male	169	77.88
	Female	48	22.22
Academic Degree	lecturer	84	38.70
	Assistant	107	49.30
	Associate	26	12.00
Years of Experience	1-5	31	14.29
	6-10	82	37.78
	11-15	53	24.43
	16-20	34	15.67
	>21	17	7.83
Faculty	Medicine	84	38.70
	Public Health	53	24.42
	Allied Medicine	38	17.51
	Nursing & Midwifery	42	19.35

Among the seven dimensions of the spiritual leadership, ‘Hope/faith’ scored the highest mean score (3.72±0.50) whilst the lowest mean was the ‘Altruistic love’ dimension (3.05 ± 0.57) (Table 2).

**Table 2:** Mean standard deviation and Kolmogorov-Shmirnov values of perceived spiritual leadership and career motivation

Variables	Dimensions	Mean	Standard Deviation	Kolmogorov - Shmirnov	p value (two-tailed)
Spiritual leadership	vision	3.40	0.62	2.33	0.12
	Altruistic love	3.05	0.57	1.51	0.24
	Hope/faith	3.72	0.50	1.72	0.40
	Meaning/calling	3.66	0.58	2.33	0.06
	Membership	3.34	0.71	1.89	0.31
	Organizational commitment	3.56	0.79	1.81	0.07
	Productivity	3.46	0.79	1.86	0.28
	**Overall spiritual leadership**	**3.45**	**0.65**	2.27	0.33
Career motivation	career identity	3.35	0.76	0.84	0.47
	Career resilience	3.53	0.44	1.32	0.06
	Career Insight	3.53	0.59	0.96	0.30
	**Overall career motivation**	**3.53**	**0.41**	1.21	0.09

The mean scores of the three dimensions of career motivation ranged between 3.53±0.41 (Table 2).

The Pearson correlation test identified a significant relationship was observed between 4 dimensions of perceived spiritual leadership (altruistic love, hope/faith, meaning/calling, membership) and career motivation and (p<0.05). According to result, between Vision, organizational commitment, productivity, and overall perceived spiritual leadership with overall career motivation was not observed any significant relationship (Table 3).

**Table 3:** Relationship between the dimensions of perceived spiritual leadership and the overall career motivation

Overall career motivation		
Perceived Spiritual Leadership dimensions	r	P-value *(two-tailed)*
Overall spiritual leadership	0.29	0.14
vision	0.28	0.07
Altruistic love	0.36	**0.01**
Hope/faith	0.47	**0.002**
Meaning/calling	0.67	**0.001**
Membership	0.46	**0.002**
Organizational commitment	- 0.01	0.91
Productivity	0.24	0.11

The independent t-test identified a significant difference in ‘Hope/faith’, ‘vision’, and ‘organizational commitment’ between males and females (P =0.04, P=0.05, P= 0.004 respectively). ANOVA revealed several significant differences in years of experience by the ‘overall career motivation’ and ‘membership’ (P=0.003, P=0.04 respectively); academic degree by ‘altruistic love’ and ‘Hope/faith’ (P=0.03); and faculty by ‘vision’, ‘altruistic love’, and ‘membership’ (P=0.03, P=0.002, P=0.04 respectively) (Table 4).

**Table 4:** Relationship between the components of perceived spiritual leadership and career motivation with demographic characteristics of the study population

Component		Gender	Years of Experience	Academic Degree	Faculty
		t	F	F	F
Overall career motivation	coefficient	2.36	4.71	0.96	0.89
	p (2-tailed)	0.12	**0.003**	0.41	0.56
vision	coefficient	8.43	0.95	0.33	8.66
	p (2-tailed)	**0.05**	0.44	0.80	**0.03**
Altruistic love	coefficient	0.36	0.76	9.87	13.34
	p (2-tailed)	0.71	0.55	**0.03**	**0.002**
Hope/Faith	coefficient	4.33	2.06	11.11	1.38
	p (2-tailed)	**0.04**	0.09	**0.03**	0.20
Meaning/calling	coefficient	0.81	0.20	1.41	0.83
	p (2-tailed)	0.37	0.93	0.24	0.61
Membership	coefficient	-0.10	7.67	0.31	1.97
	p (2-tailed)	0.91	**0.04**	0.81	**0.04**
Organizational commitment	coefficient	9.30	0.17	0.03	0.89
	p (2-tailed)	**0.004**	0.83	0.84	0.56
Productivity	coefficient	2.45	1.82	0.09	0.89
	p (2-tailed)	0.12	0.17	0.75	0.56

## 4. Discussion

Human resources in today’s educational organizations such as, universities may be facing greater stressors and psychological factors than in the past. Consequently, new organizational ways are needed to confront these factors such as attention to the spiritual dimension in leadership and the nature of the job motivation. Eventually, a participative work environment may be created which may be beneficial to the individual, the university and the community.

This study is only a correlational study that examined the relationship between the perceived spiritual leadership and career motivation among population of college educators and the differences in these variables between the subgroups of participants. The findings indicated positive relationships between altruistic love, hope/faith, meaning/calling and membership dimensions of spiritual leadership and the overall career motivation.

According to results, between Vision dimension and overall career motivation was not observed any significant relationship. If the vision is drawn more clearly and more explicitly by the leaders and clear pathways of how these goals may be achieved by the staff members, their career motivation may be enhanced. A clear vision reflects the organization units` target and the employees’ aspirations may give meaning to their work and fosters hope and faith at work. This is because the clear organizational vision enables employees to have a clear picture of the future with a clear explanation of the rationale behind the setting of such future goals (Fry et al., 2011). Similarly, Fry and Nisiewicz (2013) found that spiritual leadership may positively and significantly influence the spiritual well-being and organizational-personal performance variables by mapping a vision of faith and culture in the current progress of the organization. Eventually, the employees’ physical welfare and health may increase.

The positive relationship between career motivation and the spiritual dimension of altruism demonstrate that leaders who have a greater ability to communicate friendly with the staff and their subordinates may be more popular to the employees. This is because leaders are more able to enable transformation and enhance career motivation. A similar positive relationship was found by Winston (2002) whereby the moral and social love of the leaders considered the staff members as not only a means of achieving the organizational goals but also the employees themselves were considered as a full human beings who have needs, wants and desires which need to be met. Thus, promoting altruism in the organizations may foster deep interest in the employees’ personal current and past life which may enable them to establish good relationships with others and facilitating a network of effective relationships between individuals (Fry et al., 2005).

The existence of a positive relationship between career motivation and hope/faith in this study indicates that if the leaders will accept the organization’s commitment towards leadership roles, the employees’ motivation will be enhanced which will eventually strengthen the overall organization in today’s organizational competitiveness. Spiritual beliefs and hope/faith may serve as an inner drive in the staff members which may increase their responsibility and development (Ziyaei, Nargesian, & Aybaghi Isfahani, 2008). A belief system appears to contribute better towards the development of values, attitudes and behaviors than just merely to aspire for some specific goals.

The results demonstrated that meaning/calling of the work has a significant and a positive correlation with career motivation, which it means to feel significantly higher in the higher career motivation of educators. Pfeffer (2003) contends that feeling worthiness at work means that the defining characteristics of a profession and those of the employees do not only seek their competence and skill throughout their work, but also they give importance to the performance as a social meaning or value. Ashmos and Duchons (2000) assert that employees are always trying to have a significant inner meaningful life at work.

The significant positive relationship between the overall career motivation and membership infers that the presence of spiritual leadership in the organization may help employees to consider the importance of their job as perceived by the organizations and other stakeholders. Pfeffer (2003) views the perspective of being known as a member of the organization as including the social and cultural structures which may lead people to seek understanding and appreciation of the exposure. Fry et al. (2011) and Daft and Lengel (2000) are consistent with the results noted above and point out that being known as a member and incorporating personality in an operational network of social relationships may enhance their personal growth and development with a feeling of being more meaningful to the organization.

A positive relationship was found between the membership dimension and job motivation and individuals` work experience in the organization. If staff members’ work experience is oriented more towards the organization in which they work, they are more likely to be more motivated to do something positive about this relationship, hold organizational membership and may demonstrate a higher commitment at work.

On the other hand, a positive relationship between Altruistic love and hope/faith, and academic degree in university where educators teach, it means that the higher the academic rank of the faculty members, it is more likely that the respect to their relationships with colleagues may improve.

Spiritual Leadership form informal communication networks among individuals by altruism popular culture; this in turn may increase the sense of participation and also increase a sense of responsibility in people and all these dimensions associated with motivation.

## 5. Conclusion

Overall, the results suggest that the component of perceived spiritual leadership in higher education can increase educators` career motivation. University managers have a moral leadership power that can motivate educators, provide essential information and adequate delegation of authority, and participate them in decision-making processes and enabling them.

## 6. Limitations of the Study

The present research had some limitations such as studying only one university and a small sample as well as its limitation of results generalizability. Therefore, it is suggested that similar studies should be carried out on other public and also private university using large samples. Also, future studies should be conducted using other designs especially operation research and qualitative methods to identify and prioritize the major factors affecting on career motivation among educators in universities.

It is intended that the results of this research will help the administration sector of the university to increase the level of the educators’ job motivation by creating an infrastructure which addresses better the holistic needs of educators including their spirituality.
